# The impact of smoking on serum zinc levels among Sudanese adults: a case-control study

**DOI:** 10.11604/pamj.2026.53.145.47865

**Published:** 2026-03-27

**Authors:** Sara Sami Yousif, Tarig Hakim Merghani, Azza Osman Alawad, Shahenaz Seifaldeen Satti, Nadia Osman Yousif

**Affiliations:** 1 Department of Physiology, Faculty of Medicine, Bahri University, Khartoum, Sudan,; 2 Department of Physiology, Ras Al Khaimah College of Medical Sciences, Ras Al Khaimah Medical and Health Sciences University, Ras Al Khaimah, United Arab Emirates,; 3 Department of Physiology, Faculty of Medicine, Al Neelain University, Khartoum, Sudan,; 4 Department of Paediatrics, Ministry of Health, Khartoum, Sudan

**Keywords:** Zinc, smoking, antioxidants, tobacco

## Abstract

**Introduction:**

smoking increases oxidative stress and may impair zinc status by reducing absorption, increasing excretion, or disrupting enzyme function. Zinc is vital for antioxidant defence, and its deficiency could exacerbate smoking-related damage. This study assessed serum zinc levels in smokers and non-smokers to explore the association between smoking and zinc status.

**Methods:**

a hospital-based case-control study was conducted at Ibrahim Malik Teaching Hospital in Khartoum, Sudan. The study included 80 healthy adult males aged 18-39 years (mean age 25.56 ± 3.42 years), comprising 40 smokers as the case group and 40 non-smokers as the control group. Participants with known chronic illnesses or those on mineral supplementation were excluded. Serum zinc concentrations were measured using atomic absorption spectrophotometry. Descriptive statistics were calculated, and group comparisons were performed using the independent samples t-test. A p-value < 0.05 was considered statistically significant.

**Results:**

the mean serum zinc level was significantly lower in smokers (66.83 ± 21.13 µg/dL) compared to non-smokers (90.08 ± 14.93 µg/dL) (p < 0.05). No significant associations were observed between serum zinc levels and the number of cigarettes smoked (p = 0.683) or duration of smoking (p = 0.596).

**Conclusion:**

smokers exhibited significantly lower serum zinc levels compared to non-smokers, indicating a potential impact of smoking on zinc status. However, zinc levels did not correlate with smoking intensity or duration, suggesting the effect may occur regardless of exposure level.

## Introduction

Cigarette smoking remains one of the most significant public health challenges worldwide as it contributes to millions of preventable deaths each year [1,2]. It is widely recognised as a major risk factor for a range of systemic diseases, including cardiovascular disorders, chronic respiratory conditions, and various forms of cancer [3]. One of the primary mechanisms by which smoking exerts its harmful effects is through the induction of oxidative stress, an imbalance between the production of free radicals and the body's ability to neutralise them with antioxidants [4].

Tobacco smoke is a complex mixture containing more than 7,000 chemical compounds, many of which possess oxidising properties that contribute to cellular damage [5,6]. These compounds can generate large quantities of free radicals, which can deplete endogenous antioxidant systems [7,8]. Smoking may reduce the antioxidant pool, either due to its exhaustion or as an adaptive response resulting in imbalance and harmful effects that contribute to the pathogenesis of multiple smoking-related diseases and accelerate the ageing process. In addition, adverse effects may influence the biomolecules at the molecular level, such as lipids, proteins, and even DNA [4,9].

Cigarette smoke contains toxic trace metals such as cadmium, lead, and arsenic, which are known to exacerbate oxidative stress and disturb normal cellular function [10]. Cadmium, in particular, competes with essential bivalent metals, including zinc (Zn), especially in enzyme systems that require this cofactor for antioxidant activity [11,12]. This impairs the function of critical antioxidant enzymes such as superoxide dismutase (SOD), catalase, and glutathione peroxidase [13]. Zinc also stabilises cell membranes, modulates inflammation, and supports the nervous, cardiovascular, and immune systems. In general, zinc is involved in numerous biological processes [14,15]. It is primarily absorbed in the small intestine, and most of it is stored in muscle and bone, and only a small amount circulates in the serum, mainly bound to albumin [13,16]. It is worth noting that smoking may interfere with intestinal zinc absorption and increase zinc excretion via the kidneys or through oxidative stress-induced cellular leakage [17]. Despite adequate dietary intake, smokers may therefore experience marginal zinc deficiency (hypozincemia), making them more vulnerable to oxidative damage and its consequences [18].

Several studies have reported significantly lower serum zinc levels in smokers compared to non-smokers [18,19]. The severity of zinc depletion has been shown to correlate negatively with the number of cigarettes smoked per day and the duration of smoking history [11,18,20]. Given the critical role of zinc in antioxidant defence and the potential impact of smoking on zinc metabolism, investigating the association between smoking and serum zinc levels is of considerable clinical interest. Understanding this relationship could provide insights into the biological mechanisms underlying smoking-induced oxidative stress and inform potential nutritional or therapeutic interventions. However, there is a paucity of data on this subject among Sudanese populations, where cultural, dietary, and environmental factors may uniquely influence trace element status. Accordingly, this study aims to evaluate and compare serum zinc levels between healthy Sudanese adult smokers and non-smokers, and to examine the potential association between zinc levels and both the duration and intensity of smoking.

## Methods

**Study design and setting:** a case-control study was conducted at Ibrahim Malik Teaching Hospital in Khartoum, Sudan. The research aimed to assess and compare serum zinc levels among adult male smokers and non-smokers, using matched age groups to minimise confounding variables. Ethical approval was obtained from the Research Ethics Committee of Al-Neelain University (REC-PH-31/2022), and written informed consent was obtained from all participants before data collection. The study was conducted in accordance with the ethical principles of the Declaration of Helsinki. Participation was voluntary, and confidentiality of personal information was strictly maintained throughout the study.

**Study population and sampling:** a total of 80 apparently healthy adult males aged between 18 and 39 years participated in the study. They were equally divided into two groups: 40 smokers (case group) and 40 non-smokers (control group). All participants were employees of Ibrahim Malik Teaching Hospital, Khartoum, Sudan, to ensure feasibility, accessibility, and the ability to control environmental exposures within the two groups. Females were not included in this study to maintain a homogenous sample and reduce variability related to sex-specific physiological and behavioral differences that might confound the analysis. Participants were matched by frequency of age range, not by individual (1: 1) age matching; this is to maintain independence between the groups. Smokers were included if they had been smoking at least 10 cigarettes per day for a duration of one year or more, ensuring sufficient exposure to cigarette smoke to evaluate its potential effect on serum zinc levels. Non-smokers were selected based on having never smoked or having quit smoking more than one year before the study, with minimal or no exposure to secondhand smoke. Participants were excluded if they had acute or chronic infections, were using mineral supplements or medications that could influence zinc metabolism, or had any chronic diseases, particularly those affecting the gastrointestinal system. Before inclusion, all participants underwent clinical assessment to confirm their eligibility and ensure they met the criteria for participation in the study.

**Data collection:** after obtaining written informed consent, all participants were interviewed using a structured questionnaire designed to collect detailed information. This included sociodemographic data, smoking history such as the number of cigarettes smoked per day and the duration of smoking, as well as personal and medical history, including any medications being taken. This approach helped ensure accurate characterisation of the study population.

**Blood sample collection and zinc measurement:** participants were instructed to fast overnight for 12 hours and to refrain from smoking for at least two hours before blood collection. Venous blood samples (5 millilitres) were drawn aseptically from the antecubital vein using sterile disposable syringes and needles between 9:00 AM and 11:00 AM. The blood samples were collected in plain tubes and left undisturbed for 20 to 30 minutes to allow clotting. The samples were then centrifuged at 3000 revolutions per minute for five minutes to separate the serum. For zinc determination, 1 millilitre of reagent was added to a test tube containing 50 microliters of the serum, followed by incubation for 10 minutes. Serum zinc concentrations were measured using atomic absorption spectrophotometry on the Mindray BA-88A system.

**Statistical analysis:** all data were entered and analysed using the Statistical Package for the Social Sciences (SPSS) software, version 26.0. Descriptive statistics were used to summarise the data, including means and standard deviations, while categorical variables were presented in frequency tables. The independent samples t-test was applied to compare the mean serum zinc concentration between the smoker and non-smoker groups. Additionally, one-way analysis of variance (ANOVA) was used to assess variations in zinc concentration according to smoking intensity and duration. The Chi-square test was employed to examine the association between categorical variables, such as smoking status and serum zinc level categories. Kendall's tau-b correlation test was performed to evaluate the relationship between serum zinc level and both the number of cigarettes smoked daily and the total duration of smoking. A post hoc G*Power analysis indicated that the achieved sample size (n = 80) provided ≥80% power to detect large effect sizes in serum zinc concentrations (observed Cohen's d≈ 1.27) at α = 0.05, but was underpowered to detect small to moderate effects, particularly for subgroup analyses related to smoking intensity and duration. A p-value less than 0.05 was considered statistically significant for all comparisons.

## Results

**Participant demographics and smoking characteristics:** the study included 80 male participants, comprising 40 smokers and 40 nonsmokers, all employed at Ibrahim Malik Teaching Hospital. Participants' ages ranged from 18 to 39 years, with a mean age of 25.56 ± 3.42 years. Among the 40 smokers, 43% reported smoking fewer than 10 cigarettes per day, while the majority (57%) smoked 10 or more cigarettes daily. Regarding the duration of smoking, half of the participants (50%) had been smoking for less than 5 years, 30% for 5 to 10 years, and 20% for more than 10 years. These results suggest that while many smokers started smoking relatively recently, a substantial proportion already exhibits high daily cigarette consumption (Table 1).

**Table 1:** smoking characteristics among adult male smokers recruited from Ibrahim Malik Teaching Hospital, Khartoum, Sudan, from October 2022 to March 2023 (N = 40)

Smoking characteristic	Category	Frequency (n)	Percentage (%)
Number of cigarettes smoked per day	Less than 10	17	43%
	10 and more	23	57%
	Total	40	100%
Smoking duration	< 5 years	20	50%
	5 -10 years	12	30%
	> 10 years	8	20%
	Total	40	100%

**Serum zinc levels in smokers vs. non-smokers:** Table 2 presents a comparative analysis of serum zinc levels among participants based on tobacco use status, number of cigarettes smoked daily, and smoking duration. It shows that the mean serum zinc level in smokers was significantly lower (66.83 ± 21.14 µg/dL) compared to non-smokers (90.08 ± 14.93 µg/dL) (P<0.05). However, no significant differences between the two groups were observed based on their cigarette consumption. These findings suggest that while overall tobacco use is associated with reduced serum zinc levels, variations in smoking intensity or duration did not significantly influence zinc concentration in this study population.

**Table 2:** comparison of serum zinc concentrations by smoking status, cigarette consumption, and smoking duration among adult male participants recruited from Ibrahim Malik Teaching Hospital, Khartoum, Sudan, from October 2022 to March 2023 (N = 80)

Smoking characteristic		Number	Zinc level (µg/dL)		P-value
			Mean	SD ±	
Smoking status	Smoker	40	66.83	21.137	<0.05
	Nonsmoker	40	90.08	14.931	
Number of smoked cigarettes per day	< 10	17	65.18	22.788	0.683
	≥ 10	23	68.04	20.265	
Smoking duration	< 5 years	20	66.00	7.348	0.596
	5 -10 years	12	70.76	22.778	
	> 10 years	8	63.47	21.706	

**Zinc levels by smoking intensity and duration:** Table 3 presents the association between serum zinc levels (categorised as below or above the normal cutoff of 70 µg/dL) and smoking characteristics among the 40 smokers included in the study. Among the 40 smokers studied, 45% had serum zinc levels <70 µg/dL, while 55% had levels ≥70 µg/dL. When stratified by the number of cigarettes smoked per day, 47% of light smokers (<10 cigarettes/day) and 43% of heavy smokers (≥10 cigarettes/day) had low zinc levels. The distribution of serum zinc levels did not differ significantly between these groups (p = 0.538). Similarly, no significant association was found between smoking duration and serum zinc levels (p = 0.611); low zinc levels were observed in 50% of smokers with <5 years of smoking, 53% of those with 5-10 years, and 37% of those with >10 years of smoking history.

**Table 3:** association between categorised serum zinc levels and smoking intensity and duration among adult male smokers recruited from Ibrahim Malik Teaching Hospital, Khartoum, Sudan, from October 2022 to March 2023 (N = 40)

Smoking characteristic		Serum zinc level			P-value
		< 70 µg/dl N (%)	> 70 µg/dl N (%)	Total N (%)	
Number of smoked cigarettes per day	< 10	8 (47%)	9 (53%)	17 (100%)	0.538
	≥ 10	10 (43%)	13 (53%)	23 (100%)	
	Total	18 (45%)	22 (55%)	40 (100%)	
Smoking duration	< 5 years	2 (50%)	2 (50%)	4 (100%)	0.611
	5 -10 years	9 (53%)	8 (47%)	17 (100%)	
	> 10 years	7 (37%)	12 (63%)	19 (100%)	
	Total	18 (45%)	22 (55%)	40 (100%)	

**Correlation between smoking variables and serum zinc levels:** as shown in Table 4, Kendall's tau-b correlation analysis revealed a statistically significant negative correlation between smoking status and serum zinc levels (τ = -0.512, p < 0.05), indicating that individuals who smoke tend to have lower zinc levels compared to non-smokers. This suggests that smoking is associated with a depletion in serum zinc concentration, which may reflect increased oxidative stress or impaired nutrient absorption caused by tobacco use. However, no significant correlations were observed between zinc levels and either the number of cigarettes smoked per day (τ = 0.036, p = 0.824) or the duration of smoking in years (τ = 0.140, p = 0.366). These findings imply that while the act of smoking itself is linked to lower zinc levels, the extent or length of smoking does not show a direct dose-response relationship with serum zinc concentration in this sample. This result may be attributed to individual variability in smoking habits, nutritional status, or metabolism, and suggests that even light or short-term smoking may have adverse effects on micronutrient levels.

**Table 4:** correlation between serum zinc levels and smoking-related variables among adult male smokers recruited from Ibrahim Malik Teaching Hospital, Khartoum, Sudan, from October 2022 to March 2023 (N = 40)

Variable	Smoking status	Number of smoked cigarettes per day
Zinc level	-0.512**	0.036
P-Value	<0.05	0.824

** Kendall’s Tau-b correlation is significant at the 0.01 level (2-tailed)

## Discussion

The current study aimed to examine the impact of smoking on serum zinc levels among male hospital workers aged 18-39 years. The results demonstrated a significant reduction in mean serum zinc levels among smokers compared to nonsmokers, suggesting a clear association between smoking and zinc depletion. The mean serum zinc level in smokers was 66.83 ± 21.14 µg/dL, while in nonsmokers, it was 90.08 ± 14.93 µg/dL, with a p-value of less than 0.05, indicating a significant difference. These findings are in agreement with a growing body of evidence linking cigarette smoking to altered trace element status, particularly zinc deficiency [18].

Zinc plays a vital role in numerous physiological functions, including immune response, antioxidant defence, wound healing, and cellular growth. The reduced serum zinc levels observed in smokers may be attributed to the oxidative stress induced by smoking, which increases the demand for antioxidant defence mechanisms that rely on zinc-dependent enzymes such as superoxide dismutase [21]. Additionally, smoking increases zinc excretion through urine or perspiration. On the other hand, the heavy metals present in tobacco smoke, such as cadmium, compete with zinc for binding sites [22]. Further supporting this association, Kim *et al*. also found reduced zinc concentrations in smokers, highlighting the role of reactive oxygen species generated by tobacco combustion in accelerating the depletion of essential micronutrients [22]. Zinc is especially vulnerable to oxidative damage due to its function in regulating DNA repair and apoptosis, and chronic exposure to tobacco smoke may affect these protective mechanisms. However, not all studies are in full agreement [23]. Nevertheless, the weight of current evidence, including our findings, supports the hypothesis that smoking independently contributes to zinc deficiency.

Regarding smoking intensity, the study showed no significant differences in zinc levels between those who smoked fewer than 10 cigarettes per day and those who smoked 10 or more (p = 0.683). Similarly, the duration of smoking in years did not significantly affect serum zinc levels (p = 0.596). These findings align with the finding that the presence of smoking behavior itself was associated with lower zinc levels, while the number of cigarettes or the duration of smoking did not exhibit a clear dose-response relationship [11,20], indicating that even light or recent smoking may be sufficient to disturb zinc homeostasis, possibly due to the high toxicity and systemic effects of tobacco smoke, regardless of the exposure amount. On the contrary, other studies have found a correlation between prolonged smoking and micronutrient depletion [24]. The discrepancy between these findings and our current results may be due to sample size, population characteristics, or the narrow age range in the present study, which may limit the cumulative exposure time to tobacco toxins. It is also possible that variations in individual health status, physical activity, or nutrition could moderate the influence of smoking intensity and duration on zinc levels.

The correlation analysis using Kendall's tau-b showed a significant negative correlation between smoking status and serum zinc level (τ = -0.512, p < 0.05), indicating a moderate to strong inverse relationship. However, correlations with the number of cigarettes smoked per day and duration of smoking were not significant. These results confirm the primary finding that smoking itself, rather than the intensity or chronicity of the habit, is the main contributor to zinc depletion [25-27]. Many previous studies have shown that smokers consistently had lower serum zinc levels regardless of their smoking frequency, supporting the hypothesis that toxic components in cigarette smoke exert their effects even at low exposure levels [11,20,28].

**Limitations:** this study has several limitations that should be considered when interpreting the results. Although the case-control design is useful for identifying associations between smoking and serum zinc levels, it does not establish a temporal or causal relationship. Age was matched between the two groups based on the frequency of the age range category instead of individual age matching. This maintains the independence of the two groups; however, residual selection bias cannot be excluded. Additionally, participants were selected from hospital employees within a single healthcare setting, which may not represent the general population, as they differ from the rest of the community in their health awareness and underlying health status. As a result, the generalizability of the results can be reduced. The post hoc G*Power analysis showed that this study was sufficiently powered to detect large differences in serum zinc concentrations between smokers and non-smokers; however, it was underpowered to identify small to moderate effects, especially in subgroup analyses of smoking intensity and duration. Furthermore, because of the relatively small sample size and limited number of covariates, no multivariate regression analysis was conducted, and the possibility of residual confounding of unmeasured factors cannot be excluded. Additionally, the study did not account for other potential confounding factors, such as dietary zinc intake, alcohol consumption, or occupational exposures, that could influence serum zinc levels; thus, residual confounding cannot be ruled out. Information on smoking status and intensity was based on self-reports, which may be affected by recall bias or social desirability bias. Furthermore, biochemical markers of oxidative stress were not measured to directly assess antioxidant status. Future research should involve larger, more diverse populations and use longitudinal designs to explore the causal pathways linking smoking to micronutrient status. Future studies with a larger sample size should also apply multivariable models, which would allow adjustment for potential confounders.

## Conclusion

This study demonstrates a significant decrease in serum zinc levels among smokers compared to non-smokers, indicating that tobacco exposure can disrupt the balance of essential micronutrients. The reduction in zinc levels suggests that smoking may impair the body's antioxidant defence mechanisms. Interestingly, the findings showed no consistent relationship between zinc levels and the intensity or duration of smoking. This suggests that smoking may impair antioxidant status independent of smoking intensity, pointing to a broader systemic impact of tobacco exposure. These results underscore the need for increased awareness of the biochemical risks associated with smoking and highlight the importance of preventive strategies. Further research is needed to explore the underlying mechanisms and to evaluate whether zinc supplementation could help reduce smoking-related oxidative stress.

### What is known about this topic

- Cigarette smoking is a major source of oxidative stress, which depletes antioxidants and damages cellular components;
- Zinc is an essential trace element involved in antioxidant defence, particularly as a cofactor in antioxidant enzymes;
- Previous studies showed inconsistent findings regarding the effect of smoking on serum zinc levels, with suggestions that smoking may impair zinc metabolism.

### What this study adds

- We found that adult male smokers had significantly lower serum zinc levels compared to non-smokers in a Sudanese hospital-based population;
- We demonstrated that the reduction in zinc levels occurs regardless of the number of cigarettes smoked per day or the duration of smoking, indicating a potential threshold-independent effect;
- We provide evidence supporting the need to consider nutritional and antioxidant status in smoking-related health assessments, potentially guiding future preventive or supplementation efforts.
